# Development of competence in volumetric image interpretation in radiology residents

**DOI:** 10.1186/s12909-019-1549-3

**Published:** 2019-05-02

**Authors:** D. R. Rutgers, F. van Raamt, Th. J. ten Cate

**Affiliations:** 1 Department of Radiology, University Medical Center, Heidelberglaan 100, 3584 CX Utrecht, The Netherlands; 2 Department of Radiology, Gelre Hospitals, Albert Schweitzerlaan 31, 7334 DZ Apeldoorn, The Netherlands; 3 Center for Research and Development of Education, University Medical Center, P.O. Box # 85500, 3508 GA Utrecht, The Netherlands; 4 Radiological Society of the Netherlands, Mercatorlaan 1200, 3528 BL Utrecht, The Netherlands

**Keywords:** Clinical competence, Educational measurement, Internship and residency, Learning, Radiology

## Abstract

**Background:**

During residency, radiology residents learn to interpret volumetric radiological images. The development of their competence for volumetric image interpretation, as opposed to 2D image reading, is not completely understood. The purpose of the present study was to investigate how competence for volumetric image interpretation develops in radiology residents and how this compares with competence development for 2D image interpretation, by studying resident scores on image-based items in digital radiology tests.

**Methods:**

We reviewed resident scores on volumetric and 2D image-based test items in 9 consecutive semi-annual digital radiology tests that were carried out from November 2013 to April 2018. We assessed percentage-correct sum scores for all test items about volumetric images and for all test items about 2D images in each test as well as for all residents across the 9 tests (i.e. 4.5 years of test materials). We used a paired t-test to analyze whether scores differed between volumetric and 2D image-based test items in individual residents in postgraduate year (PGY) 0–5, subdivided in 10 half-year phases (PGY 0–0.5, 0.5–1.0, 1.0–1.5 et cetera).

**Results:**

The percentage-correct scores on volumetric and 2D image-based items showed a comparable trend of development, increasing in the first half of residency and flattening off in the second half. Chance-corrected scores were generally lower in volumetric than in 2D items (on average 1–5% points). In PGY 1.5–4.5, this score difference was statistically significant (*p*-values ranging from 0.02 to < 0.001), with the largest difference found in PGY 2.5 (mean: 5% points; 95% CI: -7.3 – -3.4). At the end of training in PGY 5, there was no statistically significant score difference between both item types.

**Conclusions:**

The development of competence in volumetric image interpretation fits a similar curvilinear growth curve during radiology residency as 2D image interpretation competence in digital radiology tests. Although residents performed significantly lower on volumetric than 2D items in PGY 1.5–4.5, we consider the magnitude of this difference as relatively small for our educational setting and we suggest that throughout radiology training there are no relevant differences in the development of both types of competences, as investigated by digital radiology tests.

## Background

Radiology residents go through several years of intensive training to learn the skills necessary for radiological image interpretation. Most of this pertains to volumetric images, as opposed to two-dimensional (2D) X-ray images that were dominant until several decades ago. Assessing image interpretation was, until then, easily done in written tests using photographs. Volumetric image interpretation requires a different approach. To assess whether residents master these volumetric skills, workplace assessments are important but also written radiology tests are still used [1–3]. Recent studies of radiology tests comprising image-based test items showed that image interpretation skills improve rapidly during the first years of residency training and level off from the 3rd to 4th training year on [3, 4]. However, a serious limitation of the radiology tests used in these studies is that all had a paper-and-pencil format, restricting them to test items with 2D images like X-ray photos or single scan slices. Volumetric radiological images, such as multi-slice computed tomography (CT) scans and magnetic resonance (MR) scans, could not be investigated in traditional tests. We do not know whether the learning curve for volumetric image interpretation develops similarly or different than for 2D images, and testing skills that do not reflect reality is undesirable. We were interested to develop a more suitable way of testing and to investigate the development of volumetric image interpretation skills.

Volumetric images consist of multiple imaging slices, which makes human-computer interactions much more prevalent in volumetric than 2D image reading. The reader of volumetric images needs to scroll through the set of multiple slices, should be able to manipulate images to optimize volumetric image interpretation and generally views anatomical and pathological findings from different directions or through various image reconstructions. As a consequence, the visual input in volumetric imaging is more dynamic, complex and extended than in 2D imaging [5–7] and the reader may need additional visual search strategies to interpret volumetric data sets [6, 8, 9]. Because volumetric image interpretation skills demand more mental effort [10], we hypothesized that competence for volumetric image interpretation may generally develop slower in radiology residents than 2D image interpretation skills. If such a systematic difference in development would exist, educators may want to adjust residency training programs.

To summarize, the purpose of the present study was to investigate how competence for volumetric image interpretation develops over time in radiology residents in digital radiology tests, and to compare this with the development of competence for 2D image interpretation.

## Methods

### Dutch radiology Progress test

The digital radiology tests that were investigated in this study were derived from the Dutch Radiology Progress Test (DRPT). The DRPT is a semi-annual comprehensive test for radiology residents in the Netherlands [11]. It is a required test for all Dutch radiology residents during their 5-year competency-based residency program, signifying a total of 10 individual tests evenly distributed over postgraduate year (PGY) 1–5. In each test, residents from all 5 PGYs participate but residents may individually apply for dispensation from participation for various reasons, such as congress attendance, holidays, leaves or circumstances in personal life. Due to these dispensation regulations and because of variations in the number of graduating and newly enrolling residents, the total number of participants varies between tests. Throughout the residency program, residents can train on a part-time basis which lengthens their training program proportionally in order to reach a net training time of 5 years at the end of the program. The DRPT has been administered since 2003, initially as a paper-and-pencil test but since 2013 in a digital format using software that has been developed specifically for image-based testing (http://vquest.bluefountain.nl/en/). The DRPT initially served a merely formative purpose, but in recent years it transitioned to a summative test. From 2017 onward all senior residents must pass the DRPT before the completion of training.

**Fig. 1:**
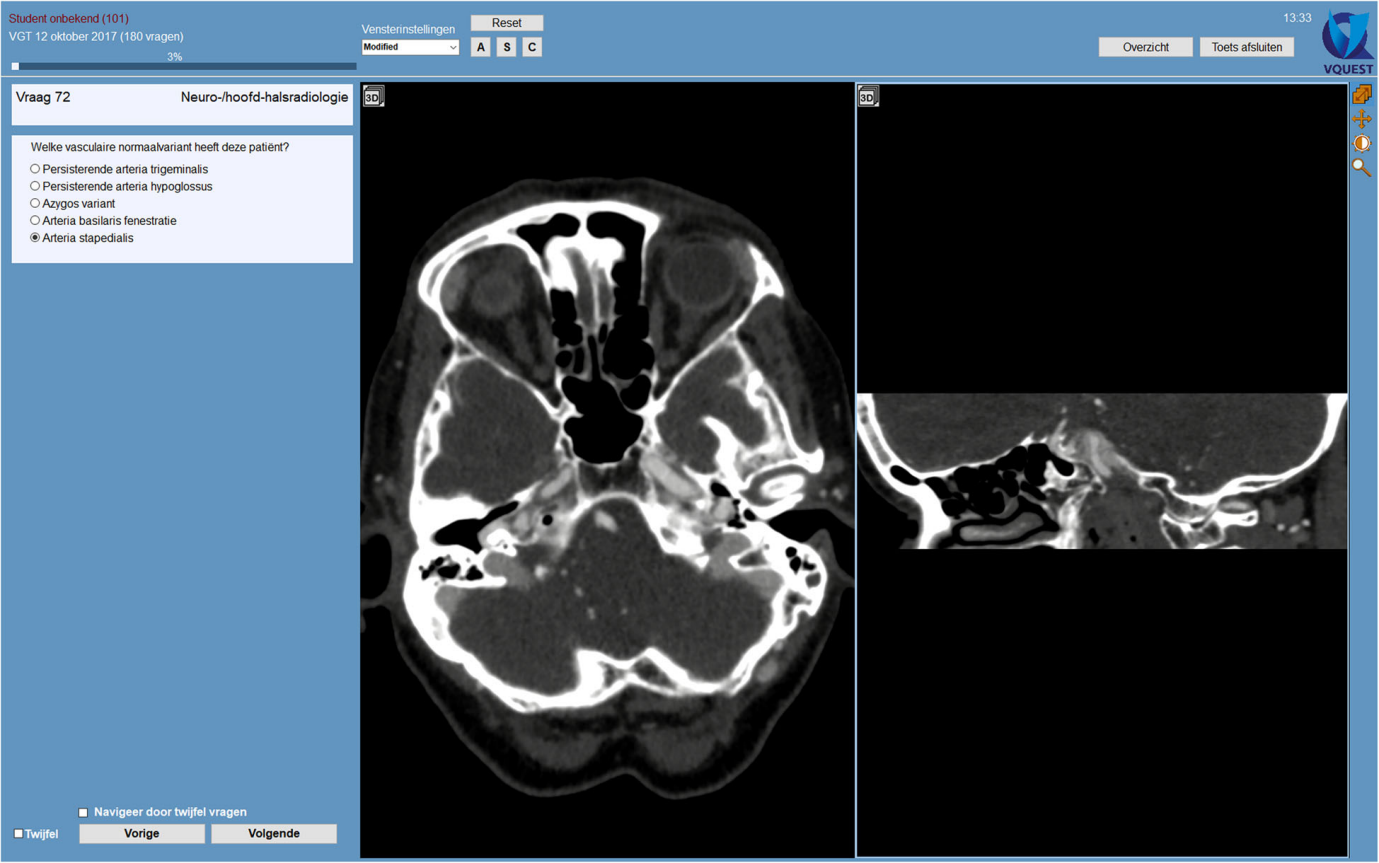
Example of a volumetric image-based test item in the Dutch Radiology Progress Test. It shows an axial volumetric computed tomography scan (left image) with a volumetric reconstruction in the sagittal plane (right image). The participant of the test can scroll through both the axial scan and the sagittal reconstruction for image interpretation. If desired, the participant can also make a reconstruction in the coronal plane (not shown). The item’s question is shown in the left panel in Dutch

The DRPT is drafted by the Examination Committee of the Radiological Society of the Netherlands and includes image-based test items, with volumetric or 2D images, and text-only items (without images). Various response formats are used, including true/false items, single right multiple choice items, drag-and-drop items and long-list-menu items. Figure 1 shows a typical example of a volumetric image-based test item in the DRPT. During the actual test, the participant can scroll through the volumetric images and can manipulate images to optimize image interpretation. After each semi-annual test, items are reviewed in post-examination test analyses, including psychometric item analysis and written item feedback from participating residents, after which the Examination Committee decides on removal of flawed items if needed, which usually is less than 5%.

### Data collection

We reviewed resident scores of 9 consecutive digital DRPTs carried out from November 2013 to April 2018. We excluded items that had been removed in post-examination test analyses of individual tests and we excluded image-based items that included schemes rather than radiological images. We categorized the image-based test items from these tests in 2 item types: volumetric image-based (items that comprised images from volumetric CT or MR scans) and 2D image-based (items that included X-ray images, ultrasound images or single slices from other cross-sectional imaging modalities, but no volumetric images). In addition, we assessed the chance of guessing a test item correctly by calculating the reciprocal of the number of answer options in that item.

For each participating resident in each individual DRPT, we calculated the percentage of correct scores on image-based test items and on the whole test. Subsequently, we combined the scores of all 9 individual DRPTs to assess overall percentage-correct score per PGY, subdividing PGYs in 10 half-year phases: PGY 0.5 indicated the residency training period from 0 to 6 months, PGY 1 the training period from 6 months-1 year, PGY 1.5 the training period from 1 year- 1 year and 6 months, et cetera. The number of PGYs reflected the net period that residents had been in training.

To account for differences in test item chance success, we calculated corrected percentage-correct scores: we first assessed for each individual DRPT the highest possible number-right score for the 2 types of image-based test items as well as for the whole test. From these highest possible scores, we subtracted the portion that participants could score correctly by mere guessing; we calculated this portion by summing the chance success scores of the individual test items concerned, in which chance success score of a given test item was calculated as the reciprocal of the number of answer options in that item. We normalized the resulting highest possible scores (i.e. highest possible scores corrected for chance success sum score) to 100 and we normalized the chance success sum score to 0. Finally, we assessed the chance-corrected number-right scores that individual residents had achieved and transposed these scores to the normalized scale. These normalized corrected percentage-correct scores of residents in a given DRPT could run from 0 to 100, but could also be negative if residents scored lower than the chance success sum score. We combined results of all 9 individual DRPTs to assess overall scores per half-year PGY phase.

### Statistical analysis

We investigated normality of parameters with the Kolmogorov-Smirnov test. We used the Mann-Whitney U-test to analyze differences in test item chance success between volumetric and 2D image-based items. We performed the Kruskal-Wallis test to investigate differences in percentage-correct score, both uncorrected and corrected for test item chance success, between PGYs for each image-based item type and for the whole test. To analyze whether corrected percentage-correct scores differed between volumetric and 2D image-based items, we performed a paired t-test making use of the pair of volumetric and 2D item scores in each individual participating resident. A *p*-value < 0.05 was considered statistically significant.

We did not perform a repeated measurement analysis on our longitudinal data because individual residents generally did not participate in each of the 9 investigated DRPTs. For example, senior residents who participated in the 2013 test did not participate in later tests because they had already finished residency at that time. Similarly, residents who newly enrolled radiology training in 2017–2018 did not participate in the 2013–2016 tests because they were not in training in these years. Also, several residents did not participate in individual tests because of dispensation. As a result, most residents in our study did not have data from all 9 consecutive DRPTs, limiting repeated measurement analysis. However, a main focus of our study was to compare volumetric and 2D item scores in residents and this could well be investigated through paired-test analysis of our data.

### Institutional review board approval

The ethical review board of the Netherlands Association for Medical Education approved this study (dossier number 1068).

## Results

Table 1 shows the number of participating residents, subdivided in 10 half-year PGY phases running from 0.5 to 5, and the number of investigated test items. The number of participating residents ranged from 316 to 367 in the 9 consecutive DRPTs (totaling 3097). A total of 1640 items, including 497 (30,3%) image-based items, was posed in the 9 tests. We excluded 15 image-based items because they had been removed after post-examination test analyses of individual DRPTs for quality reasons (*n* = 12) or because they included images other than radiological ones (*n* = 3), leaving a total of 482 image-based test items included in the present study. In addition, we excluded 53 text-only items because they had been removed after post-examination test analyses of individual DRPTs for quality reasons, leaving a total of 1572 included image-based and text-only test items.

**Table 1:** Participants and test items of the 9 Dutch Radiology Progress Tests, taken from November 2013 to April 2018, that were investigated in the present study

	DRPTs from Nov 2013 - Apr 2018	
	Range	Total
Participants (n)	316–367	3097
PGY 0.5	14–44	296
PGY 1	15–46	301
PGY 1.5	27–47	323
PGY 2	27–44	314
PGY 2.5	27–43	322
PGY 3	26–44	307
PGY 3.5	29–48	332
PGY 4	24–43	311
PGY 4.5	23–45	297
PGY 5	25–41	294
DRPT test items (n)	180–200	1640
Image-based		
Volumetric	12–25	186
2D	21–42	311
Text-only	119–163	1143
Test items included in present study (n)	171–190	1572
Image-based		
Volumetric	12–25	185
2D	20–41	297
Text-only	110–154	1090

DRPT indicates Dutch Radiology Progress Test; Nov, November; Apr, April; PGY, postgraduate year; 2D, 2-dimensional. PGY 0.5 indicates PGY 0–0.5; 1.0, PGY 0.5–1.0; 1.5, PGY 1.0–1.5; et cetera. At one occasion (October 2015) the digital test failed due to technical reasons

Test item chance scores and percentage-correct scores, both uncorrected and corrected for item chance success, were not normally distributed (*p* < 0.001, Kolmogorov-Smirnov test). The chance of guessing a test item correctly was significantly lower in volumetric test items (median 0.20 (25th percentile 0.00 - 75th percentile 0.25)) than in 2D items (0.25 (0.17–0.50); *p* < 0.001), reflecting that the former item type generally had a higher number of answer options than the latter. In the whole test, i.e. including both image-based and text-only items, median chance success was higher (0.50; 25th percentile 0.50 - 75th percentile 0.50) than in image-based items separately, due to the large contribution of text-only items that often had 2 answer options and, consequently, a relatively large chance success.

**Fig. 2:**
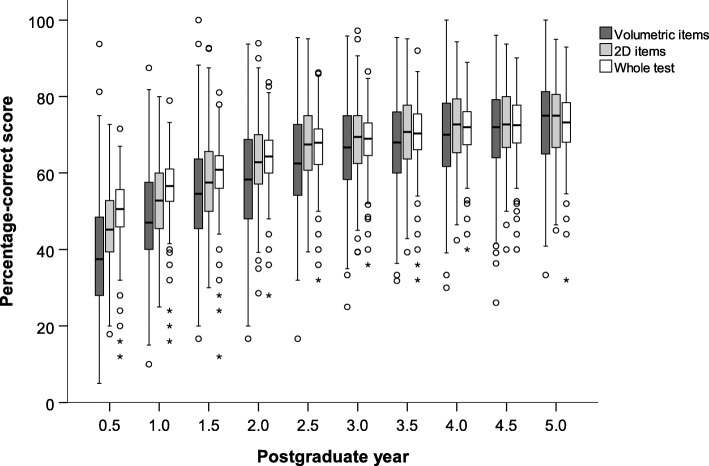
Boxplots of the residents’ percentage-correct score during 5 years of residency, divided in volumetric image-based, 2D image-based test items and whole test (i.e. including both image-based items and text-only items). Postgraduate year (PGY) 0.5 indicates PGY 0–0.5; 1.0, PGY 0.5–1.0; 1.5, PGY 1.0–1.5; et cetera

Table 2 and Fig. 2 show the percentage-correct score over consecutive PGYs for volumetric and 2D image-based test items, as well as for the whole test. Visually, the trend of development was comparable for both types of image-based test-items. In majority, scores ranged from 30 to 55% in PGY 0.5, subsequently increasing in the first half of residency and flattening off in the second half to reach approximately 65–80% in PGY 5. Both for volumetric and 2D image-based test items, as well as for the whole test, percentage-correct score differed significantly over the range of PGY phases (*p* < 0.001, Kruskal-Wallis test).

**Table 2:** Percentage-correct score during 5 years of residency, divided in volumetric image-based items, 2D image-based test items and whole test (i.e. including both image-based items and text-only items)

PGY	Items		
	Volumetric	2D	Whole test
0.5	38 (28–48)	45 (39–53)	51 (46–56)
1.0	47 (40–58)	53 (45–60)	57 (53–61)
1.5	55 (45–64)	58 (50–66)	61 (56–65)
2.0	58 (48–69)	63 (57–70)	64 (60–69)
2.5	63 (54–73)	68 (61–75)	68 (62–72)
3.0	67 (58–75)	69 (63–75)	69 (65–73)
3.5	68 (60–76)	71 (64–78)	70 (66–75)
4.0	70 (61–78)	73 (65–80)	72 (67–76)
4.5	72 (64–79)	73 (67–80)	73 (68–78)
5.0	75 (65–81)	75 (67–81)	73 (68–78)

PGY indicates postgraduate year; 2D, 2-dimensional. PGY 0.5 indicates PGY 0–0.5; 1.0, PGY 0.5–1.0; 1.5, PGY 1.0–1.5; et cetera. Data are given as median with 1st quartile-3rd quartile in parentheses

**Fig. 3:**
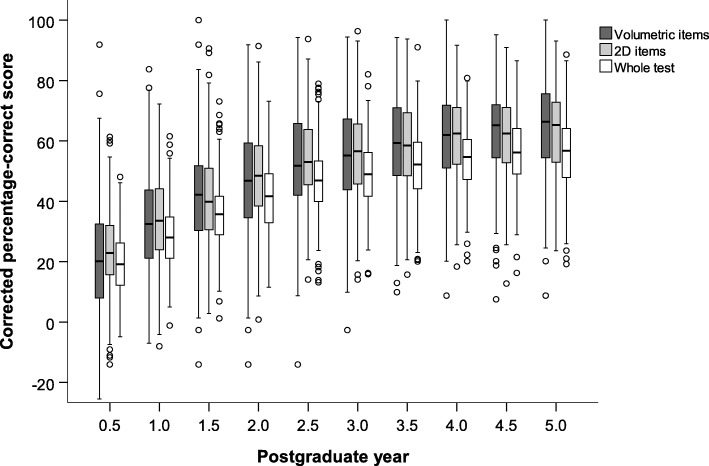
Boxplots of the normalized residents’ corrected percentage-correct score during 5 years of residency, divided in volumetric image-based, 2D image-based test items and whole test (i.e. including both image-based items and text-only items). Postgraduate year (PGY) 0.5 indicates PGY 0–0.5; 1.0, PGY 0.5–1.0; 1.5, PGY 1.0–1.5; et cetera

Table 3 and Fig. 3 show the corrected percentage-correct scores over consecutive PGYs for both types of image-based test items and for the whole test. Visually, the trend of development was comparable with uncorrected scores in Fig. 2. In the whole test, corrected scores were generally lower than in image-based items, likely due to the large contribution of text-only items that often had 2 answer options and, consequently, a relatively large chance success. Both for volumetric and 2D image-based test items, as well as for the whole test, corrected scores differed significantly over the range of PGY phases (*p* < 0.001, Kruskal-Wallis test).

**Table 3:** Corrected percentage-correct score during 5 years of residency, divided in volumetric image-based items, 2D image-based test items and whole test (i.e. including both image-based items and text-only items)

PGY	Items		
	Volumetric	2D	Whole test
0.5	20 (8–32)	23 (16–32)	19 (12–26)
1.0	32 (21–44)	34 (24–44)	28 (21–35)
1.5	42 (30–52)	40 (31–51)	36 (29–42)
2.0	47 (35–59)	48 (38–58)	42 (33–49)
2.5	52 (42–66)	53 (45–64)	47 (40–53)
3.0	55 (44–67)	57 (46–66)	49 (42–56)
3.5	59 (48–71)	58 (48–70)	52 (44–60)
4.0	62 (51–72)	62 (52–71)	55 (47–61)
4.5	65 (54–72)	62 (53–71)	56 (49–64)
5.0	66 (54–76)	65 (53–73)	57 (48–64)

PGY indicates postgraduate year; 2D, 2-dimensional. PGY 0.5 indicates PGY 0–0.5; 1.0, PGY 0.5–1.0; 1.5, PGY 1.0–1.5; et cetera. Data are given as median with 1st quartile-3rd quartile in parentheses

The difference of corrected percentage-correct scores between volumetric and 2D image-based test items in individual residents was normally distributed in each of the half-year PGY phases, allowing parametric statistical analysis. Table 4 shows the difference of corrected percentage-correct scores between both types of image-based test items. Scores on volumetric items were generally lower than scores on 2D items as demonstrated by negative values of the difference, on average ranging from − 1 to − 5% points. The score difference between both item types increased from PGY 0.5–2.5 from 0 to − 5% points. In PGY 1.5–4.5, the difference was statistically significant (*p*-values ranging from 0.02 to < 0.001), with varying confidence intervals over these years that reached a maximum negative value of − 7.3% (PGY 2.5). At the end of training (PGY 5), there was no statistical significant score difference between both item types.

**Table 4:** Difference of corrected percentage-correct score between volumetric and 2D image-based test items

PGY	Volumetric item score minus 2D item score		
	Mean ± SD	95% CI of the mean difference	*p*-value
0.5	0 ± 18	−1.9 – 2.3	n.s.
1.0	−2 ± 18	−3.8 – 0.13	n.s.
1.5	−4 ± 19	−5.9 – − 1.3	0.002
2.0	− 5 ± 19	−6.9 – − 2.8	< 0.001
2.5	− 5 ± 18	−7.3 – − 3.4	< 0.001
3.0	−2 ± 18	− 4.2 – −0.4	0.02
3.5	−3 ± 17	− 4.8 – − 1.1	0.002
4.0	−2 ± 16	−4.0 – − 0.6	0.01
4.5	−3 ± 16	− 5.1 – − 1.3	0.001
5.0	−1 ± 16	−3.1 – 0.4	n.s.

PGY indicates postgraduate year; 2D, 2-dimensional; SD, standard deviation; CI, confidence interval; n.s., not significant. PGY 0.5 indicates PGY 0–0.5; 1.0, PGY 0.5–1.0; 1.5, PGY 1.0–1.5; et cetera. Unit of ‘volumetric item score minus 2D item score’ is % points

## Discussion

The main findings of this study were twofold. First, the 5-year development of resident scores on semi-annual radiology tests showed a comparable development trend for volumetric and 2D image-based items. Second, in most half-year PGY phases (PGY 1.5–4.5) residents scored significantly lower on volumetric than on 2D items, but we consider the difference relatively small.

We found that resident scores on image-based test items in digital radiology tests, as a measure for competence in radiological image interpretation, increased in roughly the first half of residency and flattened off in the second half. This development fits the general curvilinear attainment of medical competence that is known from the literature [12, 13]. Also, it parallels radiological learning curves that have previously been derived from paper-and-pencil tests [3, 4] and digital test environments [14].

Although volumetric and 2D image interpretation skills differ profoundly, we found that competence in both skills developed quite similarly in our residents. A possible explanation for this similarity could be that answering volumetric and 2D image-based items in our radiology tests appealed to the same type of skills. To explain this, it should be noted that radiological image interpretation has both perceptual and cognitive constituents [15–18]. These can be integrated into three components of image interpretation: perception (becoming aware of something through the senses), analysis (examining the features of radiological findings) and synthesis (combining radiological and clinical findings into a conclusion) [19]. Whereas perception and analysis may differ intrinsically between volumetric and 2D image interpretation because of differences in human-computer interactions, visual input and visual search strategies [6, 8, 9], the process of synthesis may be more similar in both types of image interpretation as synthesis refers to combining radiological findings with clinical data, notwithstanding whether these findings are acquired through volumetric or 2D imaging. If synthesis processes or other image-independent skills, such as smart test-taking strategies in participants, were dominant in most of our image-based test items, this may have contributed to similarity in the development of volumetric and 2D image interpretation.

We found that percentage-correct scores that were corrected for item chance success were significantly lower in volumetric items than in 2D items in PGY 1.5–4.5. This score difference may indicate that volumetric image interpretation was more difficult than 2D image interpretation in these years. The score difference increased from PGY 0.5 to PGY 2.5, suggesting a somewhat steeper competence development on 2D items and flatter development on volumetric items in the first years of training. However, in our opinion the magnitude of the score difference was relatively small for our educational setting (the difference ranged on average from 1 to 5% points and was maximally estimated at 7.3% points based on the 95% confidence intervals). Moreover, the difference diminished in later PGYs and disappeared at the end of training in PGY 5. For these reasons, we do not consider the score difference as substantial or practically relevant for the training program as a whole. .

In competency-based medical education [20], time-variable training is a logical consequence following from the desire to graduate based on competence and not on a fixed period of time in training [21, 22]. Figures 2 and 3 show fairly large intra-individual variations in scores in all PGYs. Some residents at the end of the program (PGY 5) show scores below the majority of first year residents, which should alert educators that they may need more time in practice before completion is warranted. While scores near the end of training do not differ for image interpretation skills between volumetric and 2D proficiency, the spread among volumetric scores seems somewhat larger, across all years. It maybe worth paying attention to these skills for a specific group of residents that seem to have more difficulty.

This study has a number of limitations. First, we assessed competence for image interpretation through scores on radiology tests. These tests only cover the basic half (‘knows’ and ‘knows how’) of Miller’s pyramid-shaped framework for assessing clinical competence [23]. To confirm our results at the level of the pyramid’s top half, further study is needed in clinical simulation settings or in daily patient care. However, an examination like the DRPT with volumetric images does approach the daily work of the radiologist. Miller Level 2 (‘knows how’) using volumetric images can be considered almost a Level 3 (‘shows how’) in Miller’s pyramid, as much of what radiologists do is behind a computer screen, which is emulated in the volumetric items of the DRPT. Second, we did not investigate different cognitive processes underlying volumetric and 2D image interpretation. These processes, such as distinguished in perception and analysis [10], may have developed differently in both types of image reading. Third, as pointed out earlier, the similar development trends for volumetric and 2D item scores may indicate that both item types appealed to similar skills when residents answered them in the tests. This may suggest that the specific skills that separate volumetric from 2D image interpretation, such as scrolling-and-reading, manipulating image settings and reconstructing images, were limitedly assessed through our test items, which may ask for different item design of volumetric image-based items in future digital radiology tests.

## Conclusions

Development of competence for volumetric image interpretation fits a curvilinear growth during radiology residency and develops quite similarly compared with 2D image interpretation competence in digital radiology tests. Percentage-correct scores that were corrected for item chance success were significantly lower in volumetric items than in 2D items in various PGYs, but we consider this difference as relatively small. Our results suggest that throughout radiology training there are no relevant differences in the development of both types of competences, as investigated by digital radiology tests, and that in time-variable training focus can be put on variations in individual residents.

## Abbreviations

2D: 2-dimensional
CT: Computed tomography
DRPT: Dutch Radiology Progress Test
MR: Magnetic resonance
PGY: Postgraduate year
